# Association of Hsa-miR-23a rs3745453 variation with prostate cancer risk among Chinese Han population: A case–control study

**DOI:** 10.1097/MD.0000000000018523

**Published:** 2019-12-27

**Authors:** Minhao Zhang, Yali Wang, Can Wang, Zonghao You, Shuqiu Chen, Qingfang Kong, Bin Xu, Chunhui Liu, Ming Chen

**Affiliations:** a Surgical Research Center, Institute of Urology, Medical School of Southeast University; b Department of Urology; c Department of Nosocomial, Affiliated Zhongda Hospital of Southeast University, Nanjing, China.

**Keywords:** prostate cancer, polymorphism, miR-23a

## Abstract

Prostate cancer (PCa) is a frequently diagnosed malignant solid tumor in men. The etiology of PCa has been attributed to both environmental and genetic factors. In recent years, many studies have reported that miRNA gene single-nucleotide polymorphisms (SNPs) influence the susceptibility to several diseases such as cancer. To date, the mechanisms of PCa have remained unknown. The main aim of this study was to evaluate the association between PCa susceptibility and miRNA gene SNPs. A total of 156 PCa cases and 188 control subjects were included in this case–control study. The data were collected from hospitalized cases. We collected the demographic characteristic information, which included age, body mass index, tobacco smoking, alcohol consumption, and family history of cancer. Polymorphisms were analyzed by the ligase detection reaction. Unconditional logistic and stratified analyses were used to analyze the association between these SNPs and PCa susceptibility and to calculate the adjusted odds ratios (ORs) and the 95% confidence intervals (CIs). Cox regression model and the log-rank test were used to test the association between genetic variants and the overall survival. We found that miR-23a gene polymorphism rs3745453 carrying CC homozygotes had a 4.16-fold increased risk (95% CI = 1.30–13.25) than those carrying the TT/CT genotypes (*P* = .02), and the C allele displayed a higher prevalence of PCa than the T allele (OR = 1.68, 95% CI = 1.16–2.45, *P* = .01). Moreover, miR-23a showed that the homozygous carriers of the C-variant significantly increased the risk of survival rate as compared to the carriers of the TT/CT genotype (OR = 9.67, 95% CI = 2.83–33.09, *P* = .001). The rs3745453 polymorphism was potentially associated with PCa in the Chinese Han population and had an interactive relationship with the environmental factors.

## Abstract 2

Supplemental Digital Content is available in the text

## 1 Introduction

Prostate cancer (PCa) is the 2nd leading cause of cancer deaths in the United States. In 2018, 164,690 new cases were diagnosed with PCa, and 29,430 deaths were attributed to this disease in the United States.^[1]^ In recent years, PCa has become the 3rd common type of cancer in China, and the morbidity and the mortality of PCa have steadily increased. Thus far, the mechanisms underlying the emergence and progression of PCa have remained unknown. Previous studies have demonstrated that both genetic and environmental factors are involved in the etiology and prognosis of PCa.[^2^,^3^]

Previous studies have suggested that microRNAs (miRNAs) are involved in PCa carcinogenesis.[^4^,^5^] miRNAs are highly conserved, a class of naturally occurring, nonprotein-coding single-stranded RNAs with lengths of 21 to 24 nucleotides that can promote the degradation or inhibit the translation of target mRNAs at the posttranscription level.^[6]^ miRNAs can regulate gene expression negatively and play a crucial role in gene regulation.^[7]^ A previous study has reported that the proportion of coding protein genes regulated by the miRNAs in humans is 31%.^[8]^ There is increasing evidence that miRNAs regulate bioprocesses such as progression, cell differentiation, proliferation, and apoptosis.^[9]^

The development of PCa is an extremely complex biologic process. Under the same environment and living habits, individuals have different susceptibility to PCa. Single-nucleotide polymorphism (SNP) is formed by the variation of a single nucleotide in the genome, including transformation, transmutation, deletion, and insertion. SNP has become a 3rd-generation molecular genetic marker. Many phenotypic differences and susceptibility to drugs or diseases may be related to SNP. A large number of previous studies have shown that SNPs can not only change the type of amino acids in peptides but also affect the susceptibility of genes, treatments, and the prognosis of the disease.[^10^,^11^] The SNP in the miRNA genes can affect the maturation process and the expression level of miRNAs, leading to the occurrence and progression of a tumor. A miRNA can target hundreds of genes, and a gene can be targeted by several miRNAs.^[12]^

In recent years, many studies have reported the relationship between miRNA gene SNPs and cancer. The miRNA gene SNPs (particularly miR-146a rs2910164) posed the risk of lung cancer, colorectal cancer, and breast cancer.[^13^,^14^,^15^] Our previous studies found that several miRNAs had a series of differential expressions in PCa tissues. We hypothesized that these differential expressions were related to SNP variations. Thus far, there are no data testifying the association between miR-23a polymorphism rs3745453 and PCa susceptibility. Here, we propose that body mass index (BMI), age, tobacco smoking, alcohol consumption, and a family history of cancer may affect the modification factors for the association between the miRNA gene SNPs and PCa.[^3^,^16^,^17^,^18^,^19^] PCa DNA methylation is associated with cigarette smoking and adverse PCa outcomes,^[16]^ and alcohol consumption may increase the risk of PCa.^[17]^ Thus, in this study, we elucidated the association between miRNA gene SNPs and PCa in the Chinese Han population and further analyzed the interactive effects of genetic and environmental factors to understand the control and prevention of PCa.

## 2 Materials and methods

### 2.1 Study subjects

This study was approved by the ethics committee of Affiliated Zhongda Hospital of Southeast University. All of the patients and controls were enrolled at this hospital between January 2013 and December 2018. The PCa patients (n = 156) were diagnosed by using pathologic evidence obtained using an ultrasound-guided needle biopsy. The control group (n = 188), age-matched and without a history of cancer, was recruited during the same period from the same hospital. The excluded criteria in the control group were as follows: individuals who had an abnormal prostate-specific antigen (PSA) level or abnormal digital rectal examination. Furthermore, a 3-mL peripheral blood sample was collected with a vacuum tube, and each subject was requested to complete a questionnaire, including age, race, BMI, tobacco smoking, alcohol consumption, and a family history of cancer. According to the existing research and test investigations, smoking is one of the risk factors for PCa. The drinking habit was defined as alcohol consumption at least 3 times per week and lasting more than 10 years. A family history of cancer was defined as the presence of a malignant tumor in 1st-degree relatives (parents, siblings, and children).

Pathologic findings, pelvic computed tomography, magnetic resonance imaging (MRI), and radionucleotide bone scans were used to determine the stage of the disease. Tumor node metastasis (TNM) classification and grades were utilized to define the tumor stage according to the AJCC TNM staging system. The Gleason score was used to determine the pathologic grade. The criteria for castration-resistant PCa (CRPC) were defined according to the EAU guidelines of 2018.

### 2.2 Selection of SNPs

In our previous studies, the research findings of a chip indicated a series of differential expressions in miRNAs. Next, we searched the data by using the miRBase and dbSNP databases and selected 6 miRNA gene polymorphisms in the differential expression groups (Table 1). All the SNPs’ minor allele frequency (MAF) values were >5% in the Chinese Han population.

**Table 1:**
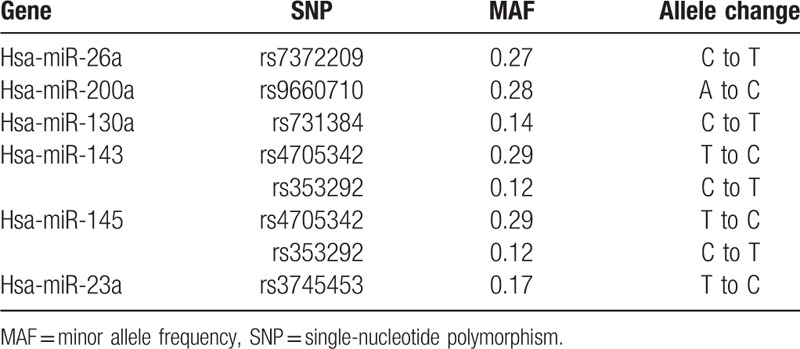
The information about selected miRNA gene SNPs.

### 2.3 Genotyping

All the genomic DNAs were extracted from the peripheral blood samples by using a TIANamp Genomic DNA kit according to the manufacturer's instructions (Tiangen Biotech Co, Ltd, Beijing, China). Polymorphisms were analyzed by polymerase chain reaction and ligase detection reaction (PCR-LDR). Each PCR was carried out in a total volume of 15 μL, which contained 1 μL of genomic DNA, 0.15 μL of each primer, 10.25 μL of H_2_O, and 3.6 μL of PCR mix (Shanghai Generay Biotechnology Co, Bio-Rad, Inc. Shanghai, China). PCR was subjected to 94°C for 3 minutes, followed by 35 thermal cycles (94°C for 30 seconds, 55°C for 30 seconds, and 72°C for 90 seconds), using a PTC 200 Thermal Cycler (Bio-Rad, Inc). The forward and reverse primer sequences are summarized in Table 2. LDR was carried out a total volume of 10 μL, which contained 3 μL of PCR products, 0.01 μL of each probe, 0.125 μL Taq DNA ligase (40 IU/μL), 1 μL Taq DNA ligase buffer. The reaction mixtures were subjected to thermal cycling using the following parameters: 94°C for 2 minutes, followed by 30 cycles (94°C for 30 seconds and 56°C for 3 minutes). One microliter of each LDR mixture was added to 8 μL of highly deionized formamide. The samples were denatured them to 95°C for 3 minutes and cooled rapidly to 4°C before loaded onto the ABI 3730XL DNA analyzer for sequencing. To determine the quality of the PCR-LDR products, 10% of the samples were regenotyped; the results obtained were consistent.

**Table 2:**
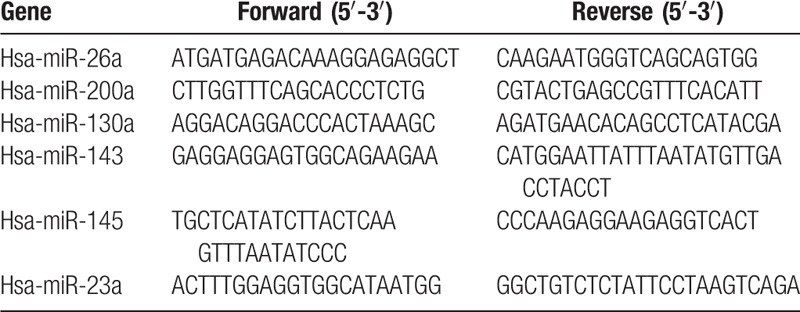
Primer sequences of microRNA genes.

### 2.4 Statistical analysis

The Hardy–Weinberg equilibrium was assessed using the goodness-of-fit Chi-squared test. A statistical analysis was performed using the statistical package SPSS 19 software (version 19.0; SPSS Inc, Chicago, IL). The distribution of demographic characteristics and genotypes was assessed by using odds ratio (OR) and 95% confidence interval (CI). A 2-sided statistical test was performed, and a *P*-value of <.05 was considered statistically significant. Unconditional logistic and stratified analyses were used to analyze the association between these SNPs and the PCa susceptibility. Cox regression model and the log-rank test were used to test the association between genetic variants and the overall survival (OS).

## 3 Results

### 3.1 Demographic characteristics of the study sample

The baseline demographic and disease characteristics were similar between the cases and the controls (Table 3), except that the proportion of the 1st-degree relatives with cancer in the case group was higher than that in the control group (26.92% vs 15.43%, *P* = .01).

**Table 3:**
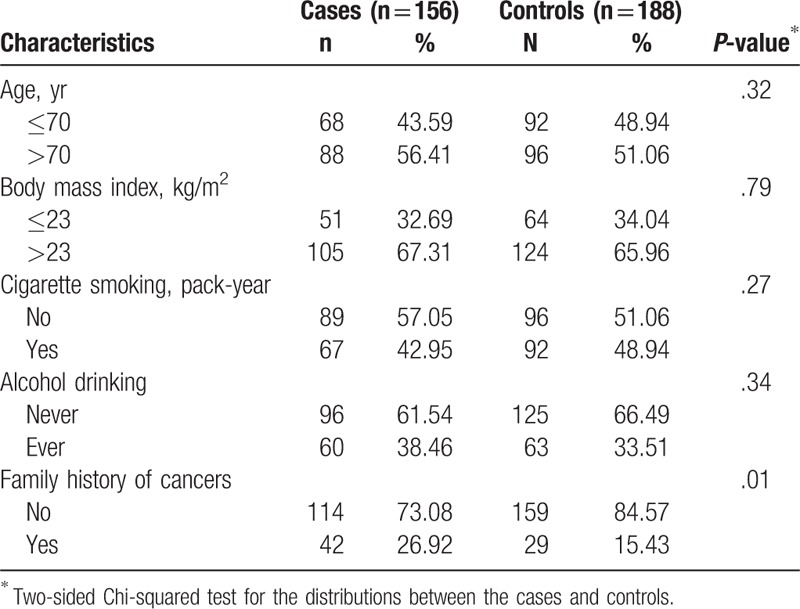
Demographic characteristic of prostate cancer cases and controls.

### 3.2 Genotype distributions of miRNAs’ polymorphism and risk of PCa

The distribution of allele frequencies in the control group was not significantly different from that of the HAPMAP CHB population. The genetic distribution of each SNP in the control group conformed with the requirement of the Hardy–Weinberg equilibrium (*P* > .05). As shown in Table 4, rs7372209, rs9660710, rs731384, rs4705342, and rs353292 were not significantly associated with PCa; however, rs3745453 was significantly associated with the PCa risk (*P* = .01). After the addition of potential covariates (age, BMI, tobacco smoking, alcohol consumption, and a family history of cancer), compared with those with the TT homozygotes, subjects carrying the CT heterozygotes (OR = 1.41, 95% CI = 0.89–2.25) and the CC homozygotes (OR = 5.23, 95% CI = 1.59–17.18) had an increased risk of PCa. In addition, subjects carrying the CC homozygotes had a 4.16-fold increased risk (95% CI = 1.30–13.25) than those carrying the TT/CT genotypes (*P* = 0.02), and the C allele displayed a higher prevalence of PCa than the T allele (OR = 1.68, 95% CI = 1.16–2.45, *P* = .01). These data showed that the allele C variation might be a risk factor for the PCa incidence.

**Table 4:**
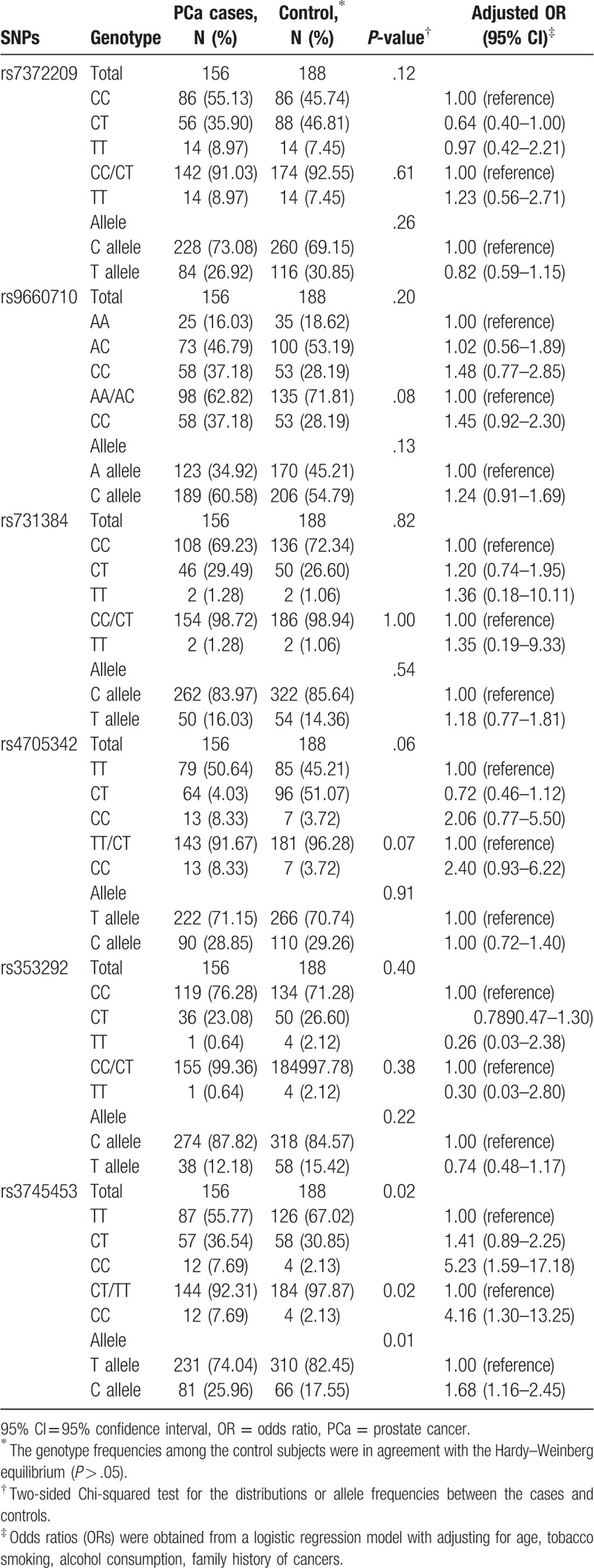
Genotypes in patients with PCa and controls.

### 3.3 Stratified analysis

After stratification by cancer stage (localized: T1–2N0M0; Advanced: T3–4NXMX or TXN1MX or TXNXM1), pathologic classification (Gleason score: <7, 7, and >7), peripheral blood PSA level (≤20, >20), and PI-RADS score of MRI (<5 and 5), we found that the higher PI-RADS score was opposite than the lower group in rs3745453 (OR = 0.46, 95% CI = 0.12–1.78, *P* = .73). As shown in Table 5, a higher Gleason score, PSA level, and disease stage exhibited the same trend for the PCa risk but no statistically significant difference. The other polymorphism loci were not statistically significant.

**Table 5:**
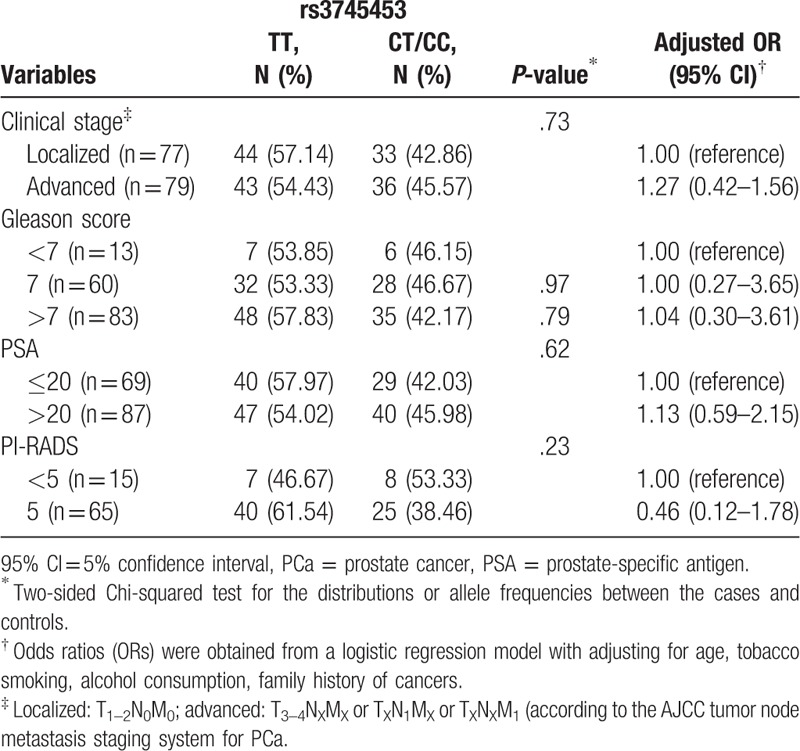
Hsa-miR-23a polymorphism and clinicopathologic characteristics in patients with PCa.

As shown in Table 6, the association between miR-23a polymorphism rs3745453 and PCa appeared more significant in the subgroups of age >70 years (OR = 1.86, 95% CI = 1.01–3.41), BMI > 23 (OR = 2.04, 95% CI = 1.17–3.58), tobacco smoking (OR = 2.22, 95% CI = 1.12–4.41), no alcohol consumption (OR = 2.04, 95% CI = 1.13–3.68), and no family history of cancer (OR = 1.83, 95% CI = 1.11–3.01).

**Table 6:**
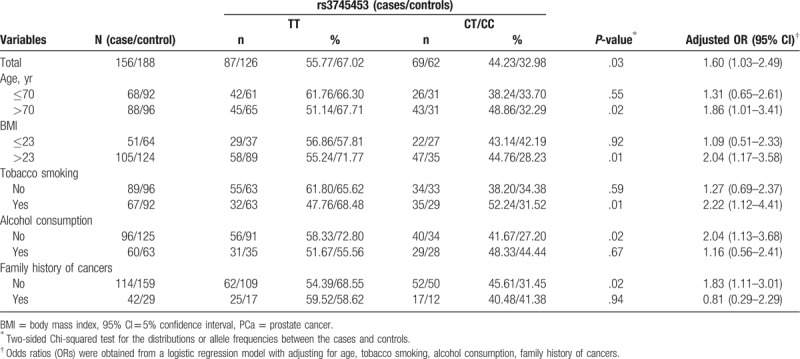
Association and stratification analysis between Hsa-miR-23a polymorphism and risk of PCa.

### 3.4 Survival analysis

**Figure 1:**
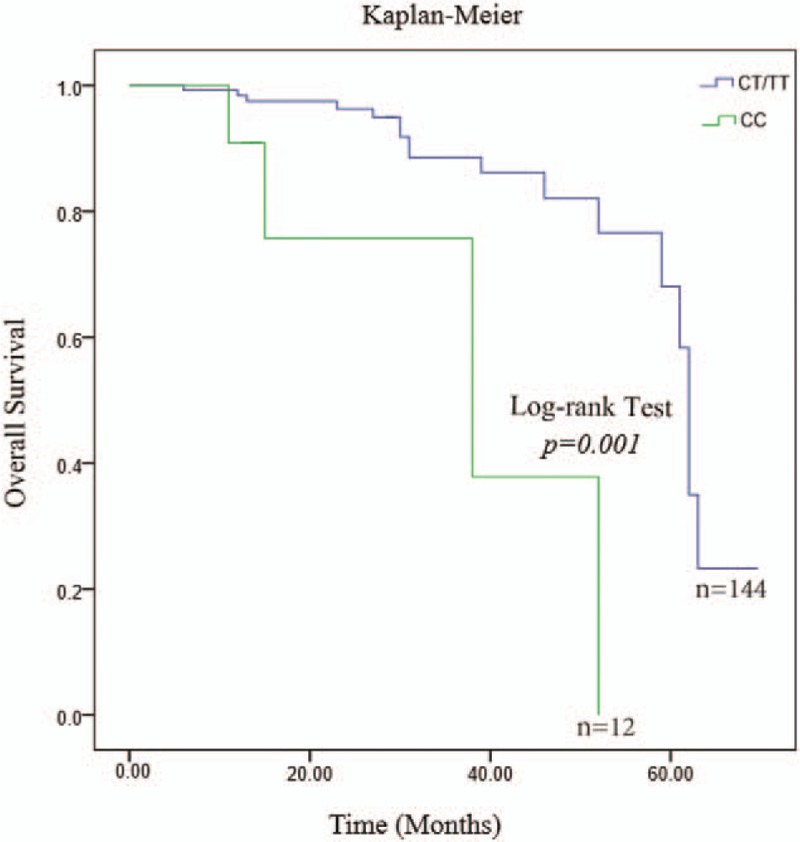
The subjects carrying the CC homozygotes had a 9.67-fold increased risk (95% confidence interval = 2.83–33.09) as compared to those carrying the TT/CT genotypes (*P* = .001) in terms of the overall survival. Cox's regression model and the log-rank test were used to test the association between genetic variants and the overall survival.

After the addition of potential covariates (CRPC occurrence time, survival time, outcome, cancer stage, age, BMI, tobacco smoking, alcohol consumption, and a family history of cancer), we found that subjects carrying the CC homozygotes had a 9.67-fold increased risk (95% CI = 2.83–33.09) as compared to those carrying the TT/CT genotypes (*P* = .001) in terms of the survival rate. As shown in Figure 1, these data showed that the allele C variation might be a risk factor for the OS.

## 4 Discussion

The studies of gene polymorphisms have opened up a widespread field for the development of clinical genetics and preventive medicine. The susceptibility of mammals to diseases can be elucidated by the correlation study between gene polymorphism and disease susceptibility, such as the study on the relationship between P53 anti-oncogene polymorphism, tumorigenesis, and metastasis, to reveal the biologic function difference among individuals from the gene level.^[20]^ Genetic susceptibility to malignant tumors has been extensively studied. Previously, researchers have demonstrated that miRNAs are involved in various crucial biologic processes through imperfect pairing with the target mRNAs of protein-coding genes.[^21^,^22^] Recent discoveries in cancer metabolomic have provided novel insights into pathways that regulate the PCa cell metabolism, with the aim of better classifying this disease and identifying new diagnostic and prognostic markers.[^23^,^24^,^25^,^26^]

We explored all the related published studies and examined their accurate locations of these SNPs. Rs7372209 is located at 2 kb upstream of miR-26a, rs3745453 is located at 3’-UTR of ZSWIM4, rs9660710, rs731384, rs4705342, and rs353292 are located at the promoter regions of their corresponding miRNAs. We found that almost all the SNPs were located at the potential functional regions, indicating that these SNPs might affect the biogenesis and expression of miRNAs.[^27^,^28^,^29^,^30^,^31^,^32^] It is attractive to propose that polymorphism in a miR-23a gene sequence may reveal a new carcinogenic mechanism. In this study, we analyzed the association between miR-23a polymorphism and the risk of PCa. We found that subjects with the CC genotype of the miR-23a gene had an increased risk for PCa compared with those carrying the TT/CT genotype. This finding sufficiently supported our hypothesis. Ridolfi et al^[33]^ reported that the allele frequency of the miR-23a rs3745453 C allele seems to act as a risk factor for multiple sclerosis. Our study demonstrated that the C variant allele might be a risk effect on the PCa incidence. In addition, we found that the increased risk associated with the CC/CT genotypes was more apparent in the subgroups as follows: subjects with age >70 years (OR = 1.86, 95% CI = 1.01–3.41) and those with a higher BMI (OR = 2.04, 95% CI = 1.17–3.58), tobacco smoking (OR = 2.22, 95% CI = 1.12–4.41), as those with no alcohol consumption (OR = 2.04, 95% CI = 1.13–3.68) and those with no family history of cancer (OR = 1.83, 95% CI = 1.11–3.01). Although the results exhibited no significant difference, as shown in Table 4, individuals who carried the CC/CT genotype were at a higher risk of PCa than those of the TT genotype with the same trend. This study suggested that the formation of PCa was associated with a variety of environmental and genetic factors. The levels of the other environmental factors were not significant, and the 95% CI included 1, but the OR value was >1, which indicated an insufficient sample size and DNA repair capacity.^[16]^ Several studies have reported that miR-23a is associated with various carcinogenesis.[^34^,^35^] Furthermore, miR-23a was associated with an increased risk for breast cancer^[34]^ and acted as an intermedium that has been sponged with LncRNA XIST to inhibit the tumor cell growth in PCa, it is proved that miR-23a promotes the progress of PCa.^[35]^ Other studies suggested that c-Myc suppression of miR-23a enhances mitochondrial glutaminase expression and glutamine metabolism, and nuclear factor-kappaB member p65 controls glutamine metabolism through miR-23a in leukemic cells.[^36^,^37^] The association between miR-23a gene polymorphism and PCa has hardly been reported in the previous studies. Several studies have reported that the variation in miRNA may affect the tumor process. For example, miR-143, a tumor suppressor of various types of human cancer, has been demonstrated to play a crucial role in tumor growth, migration, and invasion.[^38^,^39^] In the case of miR-143 polymorphism rs4705343, the T allele might be a protective factor for nonsmall-cell lung cancer in the Chinese Han population.^[40]^ The T to C variation in the miR-502 SETD8 gene increased the risk of PCa was reported by Narouie et al.^[41]^ Some of the previous studies have indicated that the association between the miRNAs gene SNPs (particularly miR-146a rs2910164) and the PCa risk was the opposite.[^42^,^43^] However, our result reported that the T to C change in miR-23a resulted in an increased risk of PCa. Some other factors such as the location of variants in the stem-loop structure or the strength of the binding between the nucleotides and different race may have caused this discrepancy. In addition, miR-23a promotes the transition from indolent to invasive colorectal cancer.^[44]^ We need further investigation of the molecular mechanisms of how genetic variants affect the PCa incidence.

## 5 Conclusion

Our findings revealed that the genetic variation in miR-23a affected the genetic predisposition to PCa and played a crucial role in the carcinogenesis. We believed that our findings opened up an opportunity and an approach to the diagnosis and therapy of PCa. In addition, we need larger, optimized prospective studies to determine this association with different ethnic samples and more environmental exposure data.

## Author contributions

**Conceptualization:** Chunhui Liu, Bin Xu, Ming Chen.

**Data curation:** Minhao Zhang, Yali Wang, Can Wang.

**Formal analysis:** Minhao Zhang, Yali Wang, Can Wang, Qingfang Kong.

**Funding acquisition:** Ming Chen.

**Investigation:** Minhao Zhang.

**Methodology:** Chunhui Liu.

**Supervision:** Bin Xu, Ming Chen.

**Validation:** Zonghao You, Shuqiu Chen, Qingfang Kong.

**Writing – original daft:** Minhao Zhang.

Ming Chen orcid: 0000-0002-3572-6886.

## Supplementary Material

Supplemental Digital Content
